# Molecular docking analysis of pyruvate kinase M2 with a potential inhibitor from the ZINC database

**DOI:** 10.6026/97320630017139

**Published:** 2021-01-31

**Authors:** Pankaj Sharma, Manvender Singh, Sangeeta Sharma

**Affiliations:** 1 Department of Biotechnology, UIET, Maharshi Dayanand University Rohtak Haryana, India; 2 School of Life Sciences, IIMT University Meerut,India

**Keywords:** Pyruvate Kinase M2, Cancer, MTiAutoDock, MTiOpenScreen, ADMET/Tox

## Abstract

The pyruvate kinase M2 isoform (PKM2) is linked with cancer. Therefore, it is of interest to document the molecular docking analysis of Pyruvate Kinase M2 (PDB ID: 4G1N) with potential activators from the ZINC database. Thus, we document the optimal molecular docking features of a compound having ID ZINC000034285235 with PKM2 for further consideration.

## Background:

Metabolism of cancer cells varies greatly from that in healthy cells [1,2,3]. Glycolysis is used in normal cells to generate energy, but glucose metabolism is transferred to aerobic glycolysis in tumour cells, and this process is called the Warburg effect [4,5]. A modern approach to targeted anti-cancer therapies is tumour glycolysis interventions [6,7,8]. Pyruvate kinase (PK) is the last rate-limiting enzyme in the glycolytic pathway, which catalyses the transfer of a phosphate group from phosphoenolpyruvate to ADP to obtain pyruvate and ATP [9,10,11]. Pyruvate kinase has four distinct subtypes [12]. In the liver, kidney and red blood cells, PKL isoforms occur mainly, whereas PKR is primarily present in red blood cells. In myocardium, skeletal muscle and brain tissue, PKM1 is distributed, and in tissues such as the brain and liver, PKM2 is distributed [13]. PKM2 is important for cancer metabolism and tumour growth, yet tetramer and dimer of PKM2 consist of the same monomer [14,15]. Between the tetramer and dimer form, biological effects are considerably different [16]. The tetramer mainly plays the role of pyruvate kinase in the sense of glucose metabolism and regulates glycolysis and dimer PKM2 as a switch for energy metabolism and material synthesis [17]. Therefore, it is of interest to document the molecular docking [18-19] analysis of Pyruvate Kinase M2 (PDB ID: 4G1N) with a potential activator from the ZINC database.

## Materials and Methods:

### Preparation of PKM2 structure:

The 3D crystal structure of Pyruvate kinase isoform M2 in complex with an activator was retrieved from the Protein Data Bank with PDB ID: 4G1N [20]. All the water molecules, Oxalate, Magnesium ions and N- (4-{[4-(pyrazin-2-yl) piperazin-1-yl] carbonyl} phenyl) quinoline-8-sulfonamide (NZT) were removed and polar hydrogen added to PKM2 protein for structure based virtual screening. Small-molecule activator NZT bind Pyruvate kinase isoform M2 at the subunit interaction interface, a site different from fructose-1, 6-bisphosphate (FBP) was used as activators binding site for molecular docking studies [20].

### Virtual screening:

The Drugs-lib library was used to conduct the virtual screening. The compound library contained 7173 approved drugs from the RPBS Web portal's in-house Drugs-lib database. The compound library was filtered by using the criteria such as molecular weight <500 Dalton; hydrogen bond donor <5, hydrogen bond acceptor <10, octanol-water partition coefficient logP <5; Number of rotatable bonds <8, polar surface area <140 Å. Docking was carried out on the MTiOpenScreen server with AutoDock Vina and with AutoDock on MTiAutoDock server [21]. To create a consensus list of compounds with both techniques that scored well, the rankings of AutoDock Vina and AutoDock were combined.

### ADME/Tox prediction:

In the drug design process, the prediction of the ADMET properties plays an important role because these properties account for the failure in the clinical phases of about 60% of all drugs. Best predicted compounds on the basis of molecular docking had been processed by PreADMET [18] tool and cross reference by Swiss ADME tool [19], which are web-based application for predicting absorption, distribution; metabolism, elimination and toxicity.

### Visualization of protein-ligand interaction:

Docking results were visualised using Python Molecular Viewer software to display docked pose of activator with PKM2 protein [22].

## Results and Discussion:

**Figure 1:**
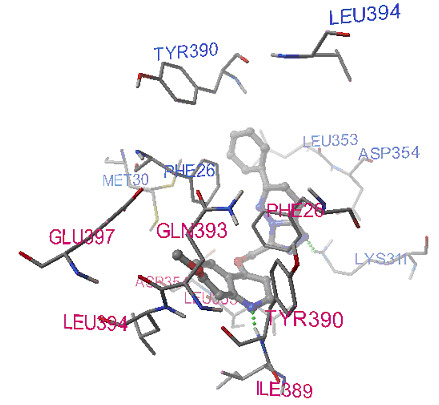
Docking pose of compound ZINC000034285235 on PKM2 protein structure. Two H-bonds were formed between amino acids TYR390, LYS311 of protein chain A and B with compound, respectively. H-Bonds are represented by green dotted spheres. Chain A and B binding residues of PKM2 protein were colored with pink and royal blue, respectively.

At the subunit interaction interface, the small molecule activator ZINC000034285235 binds PKM2, a site distinct from that of the endogenous activator fructose-1, 6-bisphosphate (FBP). PKM2 activator binding sites include 8 residues on chain A, such as TYR390, ASP354, GLN393, ILE389, PHE26, GLU397, LEU353, LEU394 and 7 residues on chain B as MET30, PHE26, TYR390, LYS311, LEU394, LEU353, ASP354. A constitutively active enzyme state (tetramer form) is promoted by PKM2 activator binding. 1500 small molecules were screened after applying filter criteria in compound library. AutoDock Vina employs a gradient-based conformational search method. The grid box parameters were set to values of 3.743 Å, -12.72, and 48.977 Å for the grid box center and 34Å x28Å x32Å for the box dimensions. We use 10 binding modes in total and 8 for exhaustiveness. The scoring of the generated docking poses and the ranking of the ligands is based on Vina's empirical scoring function approximating the binding affinity in kcal / mol. Based on their lowest binding energy, the top 100 compounds were selected and further docked with the activator binding site of PKM2 using AutoDock. Docking results of both were shown in Table 1 (see PDF). Eight compounds with ZINC000006069082, ZINC000034285235, ZINC000051951668, ZINC000000000623, ZINC000034842284, ZINC000100029945, ZINC000100031653, ZINC000064033452 were selected based on binding energy of compound with PKM2 protein having >-9.0 kcal/mol in AutoDock Vina and >-11.0 kcal/mol in AutoDock, which is even lower than control NZT compound (binding energy: -7.95 kcal/mol with PKM2 protein). Human intestinal absorption properties are essential for the production of drugs that are meant to be administered orally [23,24]. Human intestinal absorption (%HIA) values of eight drug compounds with ZINC000006069082, ZINC000034285235, ZINC000051951668, ZINC000000000623, ZINC000034842284, ZINC000100029945, ZINC000100031653 and ZINC000064033452 were shown in Table 2 (see PDF). These compounds have been identified in the category of well-absorbed compounds (HIA: 70 ∼ 100 %) [25]. In vitro cell permeability Caco-2 is an important test that measures drug intestinal absorption [26]. In the MDCK method, the cell permeability in vitro use canine kidney cells and has a shorter growth rate than the Caco-2 cells were used as a tool for the rapid analysis of permeability [27]. Skin permeability is used in the pharmaceutical industry to assess the toxicity of chemical products in the event of accidental skin contact [28]. The blood-brain barrier (BBB) is essential for drug pharmacology. PCaco-2, MDCK, Skin Permeability, PPB and BBB of eight compounds were shown in Table 2 (see PDF). Compound ZINC000034285235 had a BBB value in range 2.0 ∼ 0.1 and was graded in the central nervous system with middle absorption [29]. Carcinogenicity is the toxicity in the body that causes cancer. Compound ZINC000051951668 and ZINC000100031653 had positive carcinogenicity value in mouse and rat, respectively. Compounds ZINC000006069082, ZINC000034842284 and ZINC000100031653 had a BBB value less than 0.1 and were graded in the central nervous system with poor absorption [29]. Further, ADME properties of compounds ZINC000006069082, ZINC000034285235, ZINC000051951668, ZINC000000000623, ZINC000034842284, ZINC000100029945, ZINC000100031653 and ZINC000064033452 were analysed by SwissADME tool [19]. ZINC000051951668, ZINC000034842284, ZINC000100029945 and ZINC000100031653 were not qualified all the five different rule-based filters such as Lipinski filter implemented rule-of-five [30], Ghose [31], Veber [32], Egan [33] and Muegge [34] shown in Table 3 (see PDF). The result provided in Table 3 (see PDF) shows that all the compounds investigated have high gastrointestinal absorption except ZINC000100031653. All the compounds had good skin permeation and bioavailability score. Compounds ZINC000034285235, ZINC000000000623 and ZINC000100029945 indicate positive result in blood-brain barrier (BBB) permeation. The SwissADME estimates for passive human gastrointestinal absorption (GI) and permeation to the blood-brain barrier (BBB) consists of the BOILED-Egg model reading [35]. Therefore, only one compound ZINC000034285235 qualifies all parameters of ADME /Tox on analysis of eight best-predicted compounds using PreADMET and SwissADME tools. Best-docked complex was analyzed through Python Molecular Viewer for their interaction study shown in Figure 1. It is evident from this analysis that compound ZINC000034285235 is located at the subunit interaction interface of protein and is stabilized by hydrogen bonding.

## Conclusion:

We document the optimal molecular docking features of a compound having ID ZINC000034285235 with PKM2 for further consideration in combating cancer.
